# Modeling the Structure–Property Linkages Between the Microstructure and Thermodynamic Properties of Ceramic Particle-Reinforced Metal Matrix Composites Using a Materials Informatics Approach

**DOI:** 10.3390/ma18102294

**Published:** 2025-05-15

**Authors:** Rui Xie, Geng Li, Peng Cao, Zhifei Tan, Jianru Wang

**Affiliations:** 1 The College of Architecture and Civil Engineering, Beijing University of Technology, Beijing 100124, China; xierui0107@163.com; 2 The Institute of Xi’an Aerospace Solid Propulsion Technology, Xi’an 710025, China; ligeng102@163.com; 3 Department of Civil and Environmental Engineering, The Hong Kong Polytechnic University, Hong Kong, China; zhi-fei.tan@connect.polyu.hk; 4 Academy of Aerospace Solid Propulsion Technology, Xi’an 710025, China; wjr104zah@126.com

**Keywords:** CPRMMCs, thermodynamic properties, graph Fourier transform, principal component analysis, machine learning

## Abstract

The application of ceramic particle-reinforced metal matrix composites (CPRMMCs) in the nuclear power sector is primarily dependent on their mechanical and thermal properties. A comprehensive understanding of the structure–property (SP) linkages between microstructures and macroscopic properties is critical for optimizing material properties. However, traditional studies on SP linkages generally rely on experimental methods, theoretical analysis, and numerical simulations, which are often associated with high time and economic costs. To address this challenge, this study proposes a novel method based on Materials Informatics (MI), combining the finite element method (FEM), graph Fourier transform, principal component analysis (PCA), and machine learning models to establish the SP linkages between the microstructure and thermodynamic properties of CPRMMCs. Specifically, FEM is used to model the microstructures of CPRMMCs with varying particle volume fractions and sizes, and their elastic modulus, thermal conductivity, and coefficient of thermal expansion are computed. Next, the statistical features of the microstructure are captured using graph Fourier transform based on two-point spatial correlations, and PCA is applied to reduce dimensionality and extract key features. Finally, a polynomial kernel support vector regression (Poly-SVR) model optimized by Bayesian methods is employed to establish the nonlinear relationship between the microstructure and thermodynamic properties. The results show that this method can effectively predict FEM results using only 5–6 microstructure features, with the *R*^2^ values exceeding 0.91 for the prediction of thermodynamic properties. This study provides a promising approach for accelerating the innovation and design optimization of CPRMMCs.

## 1. Introduction

Ceramic particle-reinforced metal matrix composites (CPRMMCs) have been widely applied in various fields, such as the aerospace, automotive, and energy fields, due to their excellent physical properties, including high elastic modulus, high thermal conductivity, low coefficient of thermal expansion, and outstanding electrical conductivity [1,2,3]. Especially in the field of nuclear energy, the key properties of CPRMMCs, such as the elastic modulus, thermal conductivity, and coefficient of thermal expansion, directly influence the performance and application of materials in nuclear reactors. Although these properties are typically obtained through experimental methods, experiments are time-consuming and have certain limitations, which, to some extent, restrict the design and optimization of new nuclear reactors. Therefore, accurately predicting the thermodynamic properties of CPRMMCs in a short period has become a significant challenge in material design and optimization.

CPRMMCs are typically composed of a metal matrix and ceramic particles, with their macroscopic properties often being influenced by various factors, particularly microstructural features such as particle distribution [4,5], particle volume fraction [6,7], and particle size [8,9]. Extensive experimental studies have been conducted to evaluate the impact of microstructural features on the thermodynamic properties of composites [10,11,12,13,14]. However, experiments are often time-consuming and costly. Additionally, many theoretical models have been employed to study the thermodynamic properties of composites [15,16,17,18,19], but these models are often constrained by various assumptions and limitations, with limited predictive capability. Moreover, numerical simulations based on the finite element method (FEM) can effectively capture the linkages between microstructural features and material properties [20,21,22,23,24]. However, this method suffers from high computational costs and substantial data discretization, which constrain its applicability in large-scale optimization and rapid prediction tasks [25]. Therefore, there is an urgent need to develop a more efficient and accurate method to establish the structure–property (SP) linkages between the microstructure and thermodynamic properties of CPRMMCs.

With the development of the Materials Genome Initiative (MGI) [26] and data science technologies, data-driven approaches have provided new avenues for accelerating the study of SP linkages [27]. By mining and analyzing vast amounts of historical data and combining advanced informatics techniques, data-driven methods can accurately extract the SP linkages of materials without the need for new rounds of experiments and simulations. This approach is known as Materials Informatics (MI) [28,29,30]. Specifically, the application of this method typically relies on the establishment of “input” and “output” datasets. The “input” dataset typically includes design parameters [31,32] and microstructural features [33,34], while the “output” dataset typically contains target properties, which are obtained through experimental measurements or high-throughput computational simulations [35,36]. Next, data science techniques are used to perform statistical analysis on the datasets, simplifying the data structure by extracting and reducing the dimensionality of the microstructural features, thereby enhancing the effectiveness of the data. Common statistical methods currently used include correlation functions [37], linear path functions [38,39], and two-point spatial correlations [40,41]. Among these, two-point spatial correlations have been proven to be more rigorous and comprehensive as they can effectively capture the microstructural features of materials. As higher-order statistical datasets often contain significant redundancy and noise, dimensionality reduction techniques such as principal component analysis (PCA) [42,43,44] are widely applied to compress the data, improving its interpretability and predictive accuracy. Ultimately, through machine learning algorithms, such as support vector regression (SVR) [45,46] and convolutional neural network (CNN) [47,48], researchers can establish a mapping relationship between input data and output properties, thereby achieving accurate predictions of material properties. This data-driven integrated method not only significantly improves the efficiency and accuracy of property prediction but also effectively combines traditional research methods such as experiments, theoretical modeling, and numerical simulations, providing theoretical support and technical guarantees for the development of new materials.

In this study, an innovative method is proposed within the MI framework, integrating the FEM, graph Fourier transform, PCA, and machine learning to efficiently establish SP linkages between the microstructure and thermodynamic properties of CPRMMCs. First, the FEM is used to generate CPRMMC microstructures with different particle volume fractions and particle sizes, and their thermal conductivity, elastic modulus, and coefficient of thermal expansion are calculated. Then, the microstructural features are statistically represented through the graph Fourier transform based on two-point spatial correlations, and PCA is applied to reduce the dimensionality of the resulting statistics. Furthermore, a combination of various machine learning algorithms, including radial basis function kernel support vector regression (RBF-SVR), polynomial kernel support vector regression (Poly-SVR), random forest (RF), XGBoost, and CatBoost, is used to capture the complex nonlinear relationships between microstructural features and thermodynamic properties. The effects of different truncation levels and the number of microstructure samples on the prediction results are also systematically investigated.

## 2. Microstructure Model and Dataset

**Figure 1:**
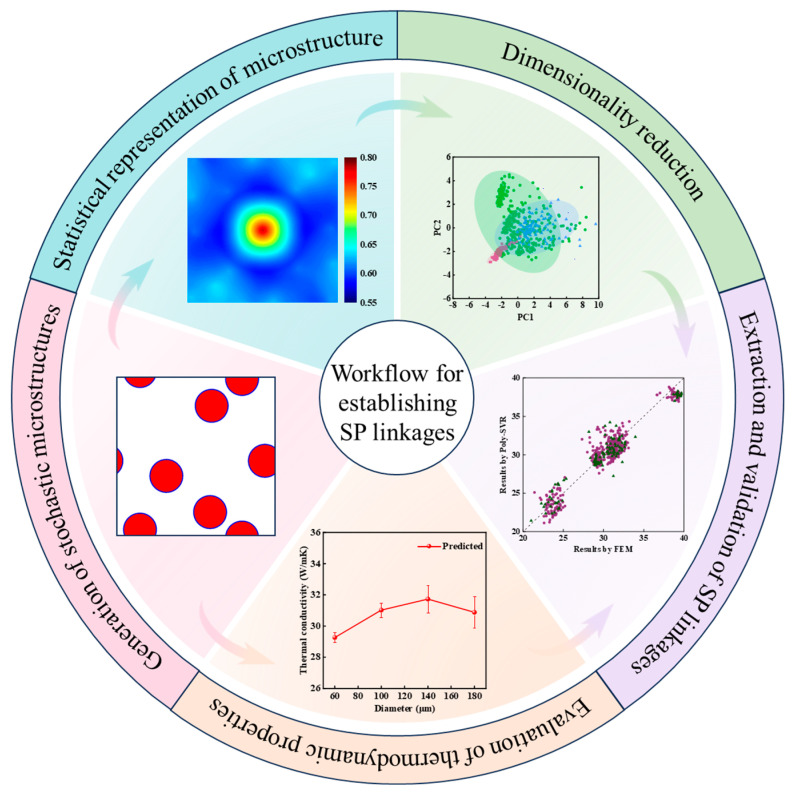
Workflow for establishing SP linkages between microstructure and thermodynamic properties of CPRMMCS.

To establish the SP linkages between the microstructure and thermodynamic properties of CPRMMCs, this study is divided into the following five steps: the generation of stochastic microstructures, the evaluation of thermodynamic properties, the statistical representation of the microstructure, dimensionality reduction of the statistics, and the extraction and validation of SP linkages. The workflow is shown in Figure 1. This section primarily introduces the methods used for generating CPRMMCs microstructures and determining thermodynamic properties.

### 2.1. Generation of Stochastic Microstructures

To explore and evaluate the capability of machine learning models in predicting the thermodynamic properties of CPRMMCs, it is first necessary to construct a dataset that reflects the fundamental facts. Due to the lack of suitable experimental datasets, this study uses FEM for simulation analysis to acquire the required dataset in a short time. The design and manufacturing process of CPRMMCs involves multiple parameters, among which the volume fraction and particle size of the reinforcing particles have a significant impact on the material’s thermodynamic properties [9,49]. Therefore, this study selects parameter combinations of different ceramic particle volume fractions and particle sizes to generate the required dataset. Although only two process parameters are used in this study to demonstrate the feasibility of establishing SP linkages through data science methods, it should be noted that the method is highly general and scalable, allowing for the inclusion of additional influencing factors to improve the model’s prediction accuracy and broader applicability.

In microstructure modeling, the size of the representative volume element (RVE) is crucial. The RVE size must be large enough to ensure that key microstructural features are included, but it should also be as small as possible to avoid excessive computational costs due to increased size [50]. Based on this, the study selects 700 μm as the RVE size, which has been shown to effectively predict the thermodynamic properties of CPRMMCs while ensuring computational efficiency [51]. For a particle size of 140 μm, particle volume fractions of 10%, 20%, and 30% are set. Meanwhile, considering the limited enhancement of thermodynamic properties at low particle volume fractions and the restrictions on thermodynamic properties at high volume fractions, the particle size is set to 60, 100, 140, and 180 μm at a 20% particle volume fraction. In summary, this study investigates six parameter combinations, with each combination generating 100 distinct particle distribution states for the RVE, resulting in a total of 600 samples.

**Figure 2:**
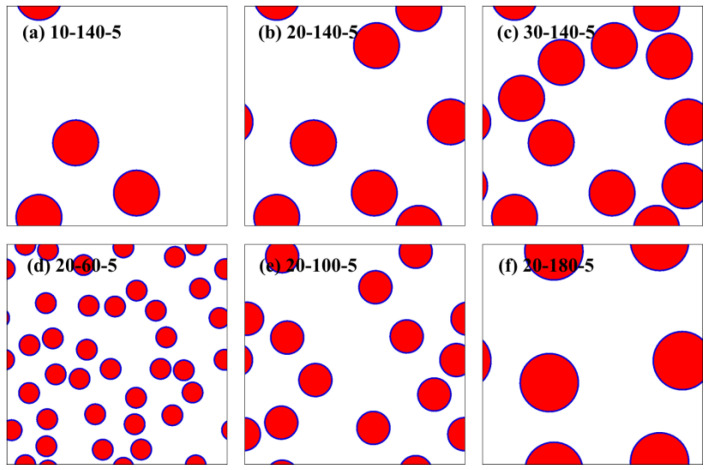
Six typical RVEs of CPRMMCs with different particle volume fractions and particle sizes.

The FEM is used in this study to create the geometric model of the RVE. The microstructure of the RVE consists of ceramic particles, the matrix, and the interface between the ceramic particles and the matrix, with the interface thickness set to 5 μm. To simplify the computational process, the ceramic particles in the model are assumed to be spherical and are randomly distributed within the RVE. This simplification effectively approximates the morphology and distribution characteristics of the particles while reducing computational complexity. Figure 2 shows six typical RVEs of CPRMMCs under different particle volume fractions and particle sizes, where red represents ceramic particles, blue represents the interface, and white represents the matrix.

### 2.2. Model Solution Method

(1) Periodic boundary conditions (PBCs)

**Figure 3:**
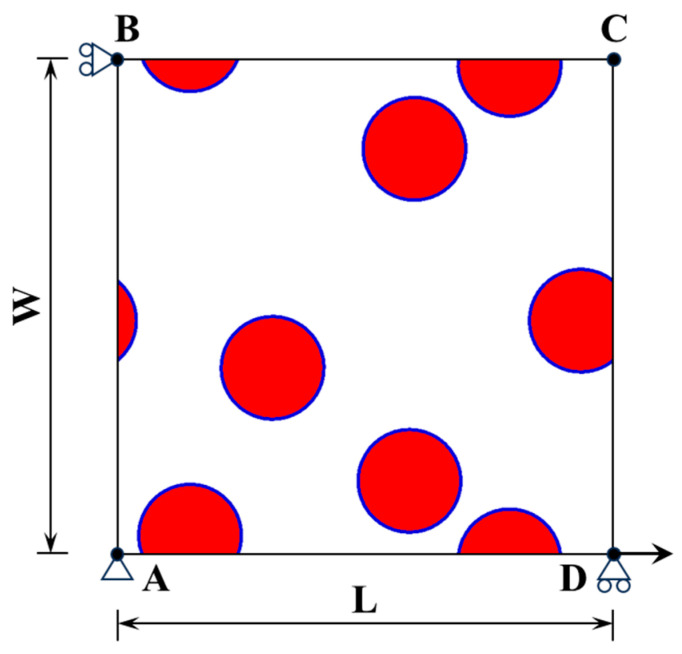
Schematic diagram of RVE with PBC.

PBCs require that the physical quantities (such as stress or temperature) at one boundary of the simulation domain are consistent with those at the opposite boundary, thereby simulating an infinite system with periodic characteristics. Specifically, consider an RVE with boundaries AB, BC, AD, and DC, where the length and width are denoted as L and W, respectively, as shown in Figure 3. The mathematical form of the PBC can be expressed as follows [52]:

(1) $$\begin{array}{l} u_{BC}-u_{B}=u_{AD}-u_{A}\\ u_{AB}-u_{A}=u_{DC}-u_{D} \end{array}$$

where *u_AB_*, *u_BC_*, *u_AD_*, and *u_DC_* represent the displacement vectors of arbitrary material points on the corresponding boundaries, and *u_A_*, *u_B_*, *u_C_*, and *u_D_* represent the displacement vectors at each vertex. Strain is generated by applying horizontal displacement at vertex C to calculate the elastic modulus of CPRMMCs.

(2) Thermal conductivity

In thermal conductivity analysis, the heat conduction equation is one of the fundamental equations used for understanding and describing how heat propagates through a material. The distribution of heat flux *q* typically follows Fourier’s law, which states that heat flux is a function of the temperature gradient and can be expressed as follows:

(2) q=−λ∇T

where *λ* represents the thermal conductivity coefficient, a constant that measures the material’s ability to conduct heat, and ∇*T* is the temperature gradient, indicating the direction of heat flow from higher to lower temperature regions. In this study, the steady-state heat flux of the RVE under different temperature conditions is calculated using the FEM, and then the thermal conductivity is determined using Equation (2).

(3) Coefficient of thermal expansion

**Figure 4:**
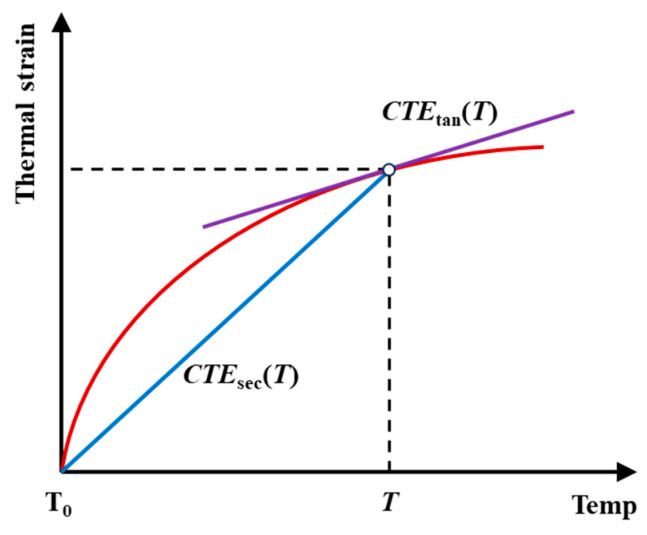
Calculation method of CTE under different temperature conditions.

The coefficient of thermal expansion (CTE) of CPRMMCs is determined by studying the effect of temperature changes on the material’s strain. When using the FEM to calculate the CTE, the thermal expansion process needs to be modeled as the effect of temperature on the material’s properties. Specifically, by defining the relationship between the time step and temperature, the heating process is simulated to obtain the material’s thermal strain–temperature curve, as shown in Figure 4. In this figure, *CTE*_sec_ represents the secant coefficient of thermal expansion, *CTE*_tan_ represents the tangent coefficient of thermal expansion, and *T* denotes any given temperature. Typically, the heating process at 1000 °C is divided into 100 equidistant incremental steps for computation. This method can accurately reflect the impact of temperature changes on the thermal expansion characteristics of the composite material and provides effective data support for material design.

In the actual calculation, the following steps are used: (1) define the relationship between temperature and elastic modulus; (2) apply temperature loading from the reference temperature to the maximum temperature; (3) compute the thermal deformation in the RVE; (4) calculate the effective thermal strain; (5) calculate the *CTE*_sec_ at different temperatures.

### 2.3. Evaluation of Thermodynamic Properties

In this study, UO_2_ material is used as the ceramic particle, and Zr alloy is used as the metal matrix. The material properties of the ceramic particles, metal matrix, and interface are shown in Table 1.

When using the FEM for simulation analysis, the model is meshed using triangles. A mesh sensitivity analysis was conducted to ensure that the chosen mesh density provides accurate results without unnecessary computational costs. The mesh size was gradually refined, and the solution was found to converge when further refinement led to negligible changes in the results. To balance accuracy and computational efficiency, the phases in the model used for calculating the elastic modulus and thermal conductivity are meshed into approximately 15,000 elements and 10,000 nodes, while the phases in the model used for calculating the CTE are meshed into approximately 150,000 elements and 80,000 nodes. Additionally, CPRMMCs can be considered as a periodic array of RVEs, so PBC must be applied to the RVE. This means that each RVE in CPRMMCs exhibits the same deformation pattern, and there is no separation or overlap between adjacent RVEs.

**Figure 5:**
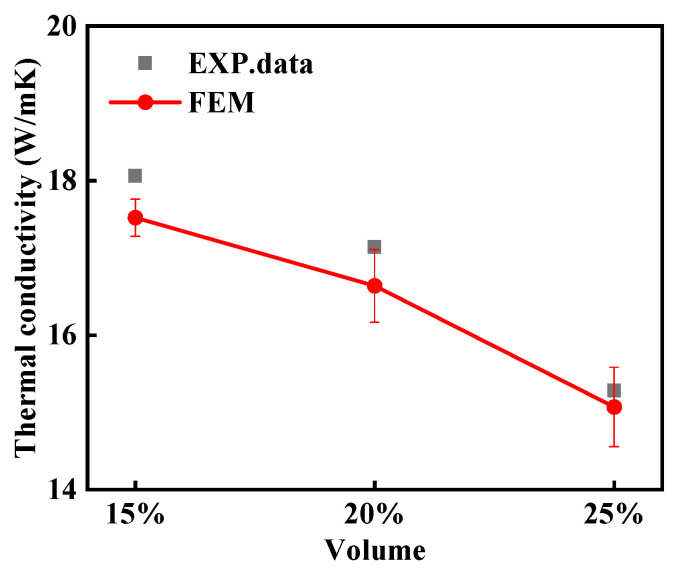
Comparison of simulation and experimental results for thermal conductivity at different particle volume fractions.

To verify the reliability of the finite element simulation, the simulation results for thermal conductivity at different particle volume fractions are compared with the experimental results, as shown in Figure 5. From the figure, it can be seen that the experimental and simulation results are in good agreement. This indicates that the established micro-mechanical model is reliable and can be used for the subsequent establishment of SP linkages.

**Figure 6:**
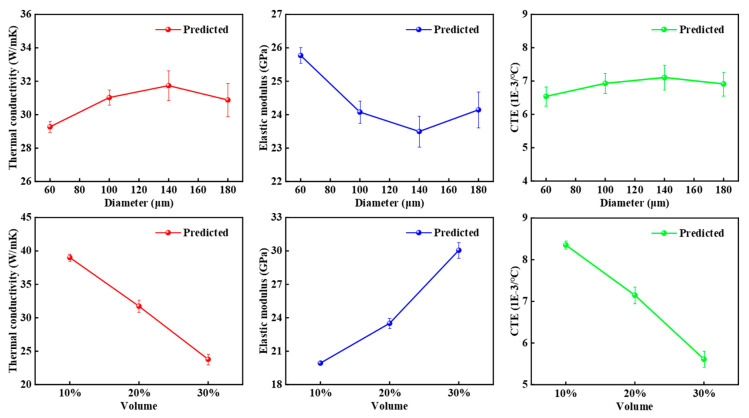
Statistical results of thermal conductivity, elastic modulus, and CTE under different particle sizes and volume fractions.

Figure 6 shows the statistical analysis of thermal conductivity, the elastic modulus, and the CTE for 600 RVEs. From the figure, it can be observed that as the particle size increases, thermal conductivity and the CTE initially increase and then decrease, reaching a maximum value of 140 μm. The elastic modulus first decreases and then increases, reaching a minimum value of 140 μm. As the particle volume fraction increases, thermal conductivity and the CTE decrease, while the elastic modulus increases. This indicates that both the particle size and particle volume fraction have a significant impact on the thermodynamic properties of CPRMMCs.

## 3. Microstructure Dimensionality Reduction and Machine Learning Methods

### 3.1. Statistical Representation of Microstructure

**Figure 7:**
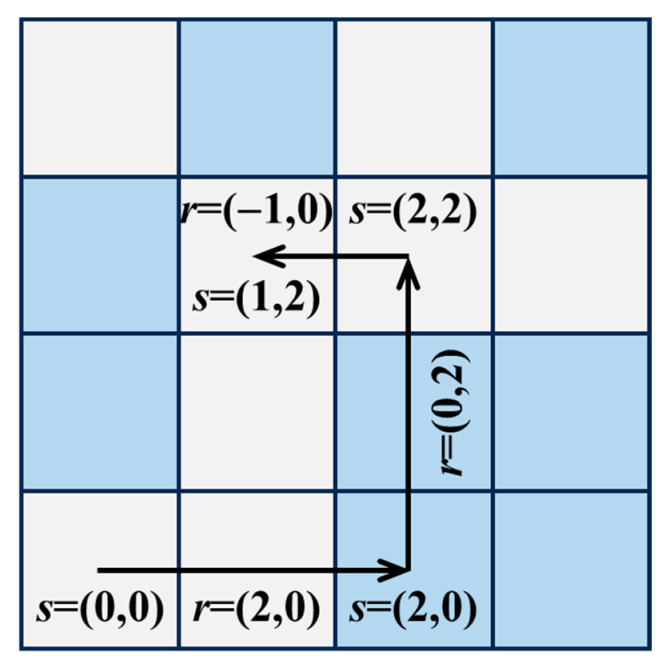
Illustration of discretized microstructure $m_{s}^{h}$ [41].

The concept of two-point spatial correlations involves treating the microstructure image as a matrix containing positional and state information. By calculating the correlation between the state of each position in the matrix and other positions, the correlation characteristics of the entire microstructure image can be statistically analyzed. To facilitate this calculation, it is necessary to discretize the material’s microstructure, which involves dividing the continuous microstructure into distinct regions, each characterized by a uniform local state. The discretized microstructure is then represented using a functional expression. This discretized microstructure includes the spatial positions (denoted by vector *s*) and the local states (denoted by *h*) across the entire spatial domain. The two-point spatial correlations can be expressed as a function [55]:

(3) $$f_{r}^{hh^{\prime }}=\frac{1}{S_{t}}\sum_{s=1}^{S_{t}}m_{s}^{h}m_{s+r}^{h^{\prime }}$$

where *s* represents a spatial point within the entire microstructure domain *S_t_*. In this study, *S_t_* refers to all pixel points in the microstructure image, while *s* represents the spatial location coordinate of each pixel. $m_{s}^{h}$ represents the probability density of finding a local state *h* at spatial position s [56]. That is, if the local state at position *s* is *h*, the probability density is 1, and if the local state at s is any other state, the probability density is 0. Based on this, two-point spatial correlations can be expressed as follows: a vector *r* connects two spatial points, *s* and *s* + *r*, and the probability density of finding structural states *h* and *h*′ at these two points is computed. As shown in Figure 7, by varying the vector *r*, this calculation is extended across the entire spatial domain and summed [57]. The expression $f_{r}^{hh^{\prime }}$ in Equation (3) represents the cross-correlation of *h* and *h*′, which calculates the correlation of different local states, while $f_{r}^{hh}$ represents the auto-correlation of *h* and *h*. In general, when *S_t_* is large, higher-order calculations are required, and the computational process becomes particularly complex. Previous studies have shown that the Fast Fourier Transform (FFT) can reduce the computational complexity from *O*(*N*^2^) to *O*(*N*log*N*), making the Fourier transform of large-scale data more efficient [58,59,60]. To speed up the computation, this study employs FFT to calculate Equation (3).

In this study, two-point correlation statistics are applied to the microstructure images of CPRMMCs. Each image is discretized into 692 × 692 pixels, resulting in a total of *S_t_* = 478,864 spatial points. The matrix, interface, and ceramic particles are labeled as 0, 1, and 2, respectively, meaning each pixel belongs to one of three local states, i.e., *h* ∈ {0,1,2}. Accordingly, there are nine possible types of spatial correlations, denoted as $f_{r}^{hh^{\prime }}$, including three auto-correlations (where *h* = *h*′) and six cross-correlations (where *h* ≠ *h*′). Since the matrices calculated for $f_{r}^{01}$ and $f_{r}^{10}$, $f_{r}^{02}$ and $f_{r}^{20}$, and $f_{r}^{12}$ and $f_{r}^{21}$ are the same, and the volume fraction of the interface in the microstructure images is negligible, this study focuses only on three representative types of correlation: matrix auto-correlation $f_{r}^{00}$, particle auto-correlation $f_{r}^{22}$, and matrix–particle cross-correlation $f_{r}^{02}$.

**Figure 8:**
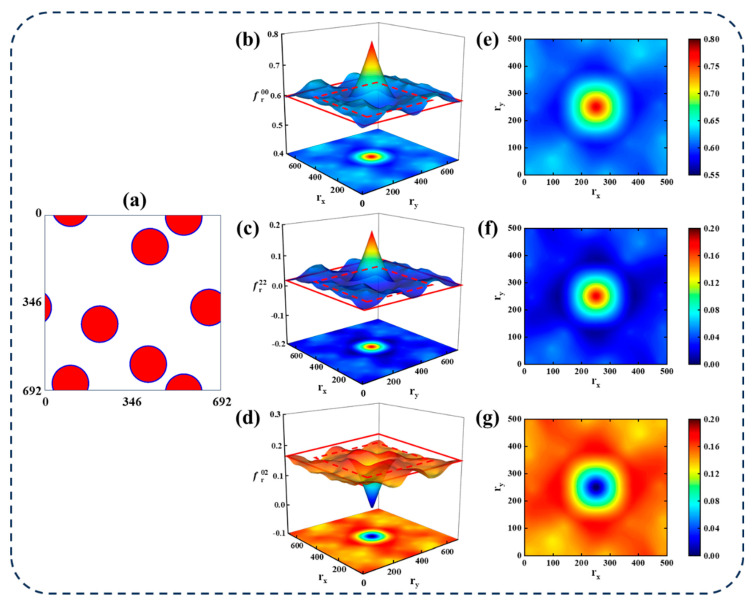
Statistical representation of CPRMMC microstructure. (**a**) Microstructure image. (**b**–**d**) Three-dimensional representations of two-point statistics and truncation settings for (**b**) matrix auto-correlation, (**c**) particle auto-correlation, and (**d**) matrix–particle cross-correlation. (**e**–**g**) Two-dimensional representations of truncated two-point statistics for (**e**) matrix auto-correlation, (**f**) particle auto-correlation, and (**g**) matrix–particle cross-correlation.

The microstructure used in the finite element model for thermodynamic property calculations of CPRMMCs is shown in Figure 8a, with an image resolution of 692 × 692 pixels. Figure 8b–d display the three-dimensional mappings of the matrix auto-correlation, particle auto-correlation, and matrix–particle cross-correlation, respectively. It is important to note that the *x* and *y* axes represent the spatial locations of the two-point correlation statistics, while the *z* axis denotes the magnitude of the statistical values.

As observed in Figure 8b,c, the center of each auto-correlation plot (where the vector *r* = 0) corresponds to the volume fraction of the matrix and particles, respectively, and the amplitude of the surrounding fluctuations is approximately equal to the square of the respective volume fractions. In contrast, the center value of the cross-correlation plot (Figure 8d) is zero as it is physically impossible for both the matrix and particle phases to coexist at the same spatial location. The amplitude of the surrounding fluctuations in the cross-correlation plot is approximately equal to the product of the matrix and particle volume fractions. Previous studies have shown that the dominant microstructural features are primarily concentrated within the region corresponding to small vector values in the statistical data. Therefore, to simplify subsequent analyses, only a subset of the statistical data is retained. The truncated regions, highlighted by red dashed boxes in Figure 8b–d, correspond to the preserved two-dimensional slices shown in Figure 8e–g. Specifically, 96 columns of statistical values are truncated from both the x and y directions. As a result, the total data size is reduced from 3 × 692^2^ = 1,436,592 to 3 × 500^2^ = 750,000, which significantly enhances computational efficiency in the following steps. The factor of 3 accounts for the three types of correlation used to represent the microstructure: matrix auto-correlation $f_{r}^{00}$, particle auto-correlation $f_{r}^{22}$, and matrix–particle cross-correlation $f_{r}^{02}$. The influence of different truncation levels will be further discussed in a later section.

**Figure 9:**
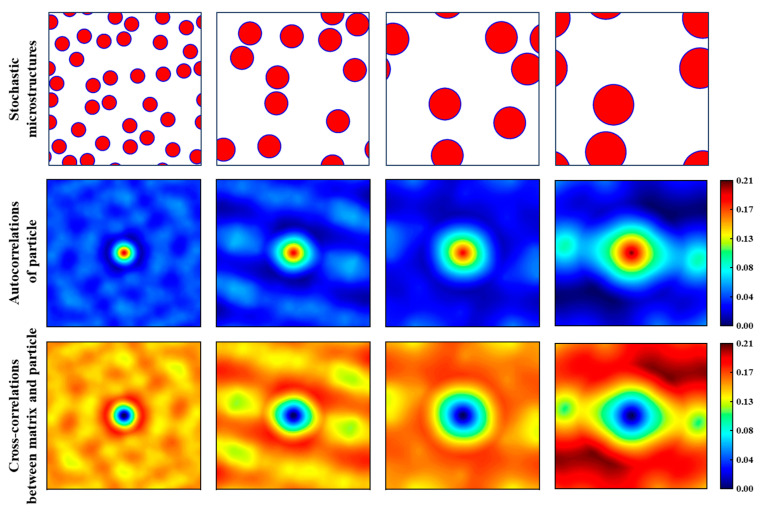
Four typical RVEs with particle sizes of 60, 100, 140, and 180 μm and their corresponding two-point statistics for particle auto-correlation and matrix–particle cross-correlation.

Furthermore, Figure 9 illustrates the two-point statistics of particle auto-correlation and matrix–particle cross-correlation at a constant particle volume fraction of 20%, with particle sizes of 60, 100, 140, and 180 μm. As shown, for the particle auto-correlation map at a particle size of 60 μm, a bright circular region appears at the center, while the surrounding area is filled with numerous small bright spots. As the particle size increases, both the diameter of the central bright circle and the size of the surrounding bright spots increase accordingly. A similar trend is observed in the matrix–particle cross-correlation maps. This phenomenon may be caused by the smaller size and larger number of small-sized particles than large-sized particles, which indicates that two-point spatial correlations statistics are capable of effectively capturing the characteristic features of the microstructure.

### 3.2. Dimensionality Reduction of Statistics

Although two-point spatial correlations can effectively capture key features within the microstructure, the challenge of high dimensionality remains. Even after dimensionality reduction through large vector truncation, the representation of microstructures remains excessively high-dimensional, making it difficult to establish effective SP linkages in practical applications [61]. This high-dimensional representation not only leads to significantly increased computational resource requirements but may also reduce the model’s generalization capability due to data sparsity, thereby compromising prediction performance. PCA has been proven to provide a reliable and accurate low-dimensional representation of high-dimensional spatial correlations [62,63]. As a classical unsupervised learning technique, PCA reconstructs the feature space through orthogonal linear projection and ranks the principal components (PCs) in descending order according to their explained variance, ensuring that the first principal component retains the most relevant information from the original data. In addition, PCA introduces orthogonal basis vectors, which successfully decouple the nonlinear correlations among the original features, providing a solid mathematical foundation for constructing efficient and stable SP prediction models.

In this study, the two-point spatial correlations statistics of microstructures are projected into the principal component space to achieve dimensionality reduction. The vectorized representation of the *k*-th microstructure in the PC space is given as follows [57]:

(4) $$f_{r}^{\left(k\right)}=\sum_{i=1}^{\min\left(\left(K-1\right),R\right)}\alpha_{i}^{\left(k\right)}\varphi_{ir}+{\bar{f}}_{r}$$

where *K* denotes the total number of microstructures, *R* represents the retained dimensionality of the two-point spatial correlations statistics, $\alpha_{i}^{\left(k\right)}$ is the weight coefficient of the *k*-th principal component, $\varphi_{ir}$ refers to the basis vectors in the transformed space, and ${\bar{f}}_{r}$ is the mean of the reduced dataset.

By performing singular value decomposition (SVD) on the original data matrix *X*, the following decomposition can be obtained:

(5) $$X=U\Sigma V^{T}$$

where the basis vectors $\varphi_{ir}$ correspond to the matrix *V^T^*, while the PC weight coefficients $\alpha_{i}^{\left(k\right)}$ are derived from the relevant portion of the matrix *U*Σ. It is noteworthy that the first principal component typically represents the direction of greatest variance in the data and is associated with the largest eigenvalue. By retaining a reduced number of principal components (i.e., *R*′ PCs), the dimensionality-reduced representation of the *k*-th microstructure can be approximated as follows:

(6) $$f_{r}^{\left(k\right)}\approx \sum_{i=1}^{R^{\prime }}\alpha_{i}^{\left(k\right)}\varphi_{ir}+{\bar{f}}_{r}$$

It should be noted that the number of principal components to retain is typically determined by calculating the proportion of variance explained (PVE), which reflects the ratio of variance explained by each principal component to the total variance of the dataset. PVE helps assess the relative importance of each principal component and guides the selection of the number of components to retain based on the desired level of accuracy. Fewer retained components result in lower dimensionality, improving computational efficiency while preserving the essential information of the data.

**Figure 10:**
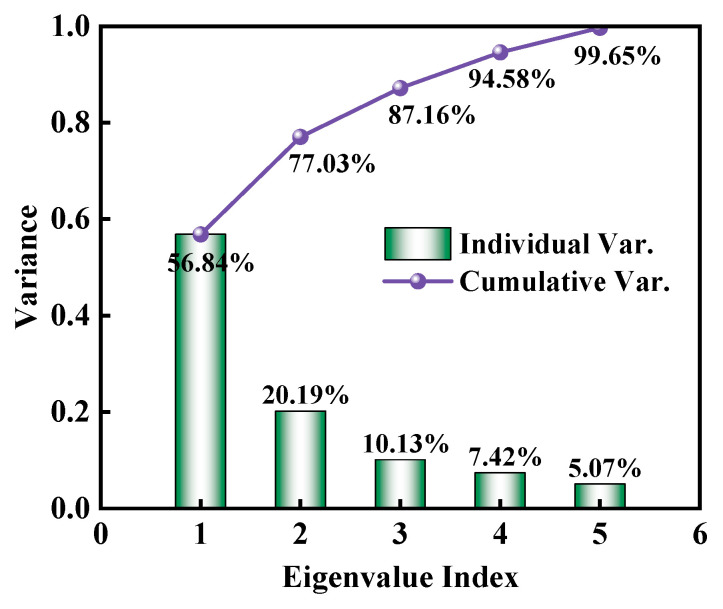
Scree plot of first five principal components.

The high-dimensional feature vector space obtained from the two-point spatial correlations statistics of microstructure images is reduced using PCA. The variance explained by each PC is illustrated in Figure 10. In this figure, the variance contributed by each individual principal component—corresponding to a specific microstructural feature—is represented by bars, while the cumulative variance from the first PC to the selected number of PCs is shown as a curve. As observed in the figure, the cumulative PVE by the first three principal components exceeds 87%. In other words, the first three PCs capture the vast majority of the microstructural features represented by the two-point spatial correlations statistics. With the addition of a fourth PC, the cumulative PVE surpasses 94%. This indicates that the dimensionality of the dataset is effectively reduced from 750,000 to just 4 while retaining nearly all critical information. These results demonstrate the remarkable capability of PCA to achieve the high-precision dimensionality reduction in two-point spatial correlations features for microstructure representation.

**Figure 11:**
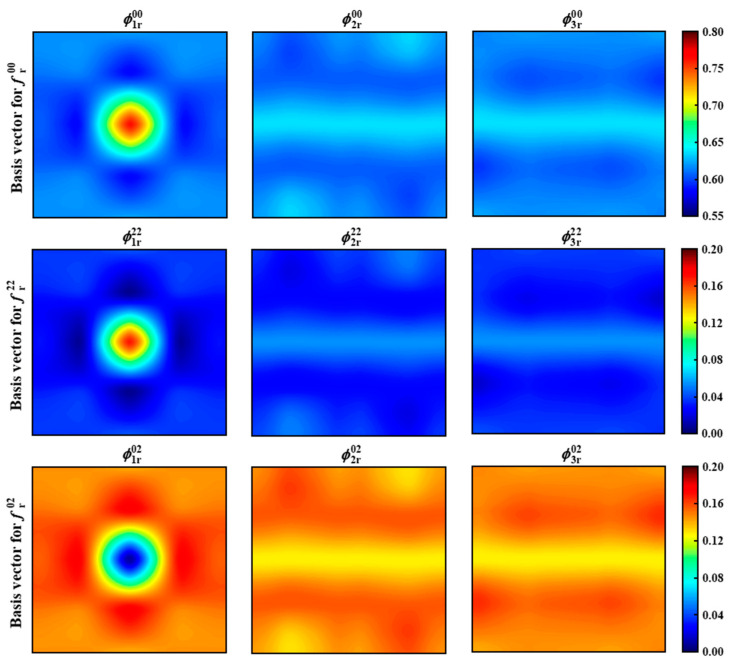
The first three PC basis vectors of the two-point statistics for matrix auto-correlation, particle auto-correlation, and matrix–particle cross-correlation.

Figure 11 presents the first three basis vectors of the two-point spatial correlations statistics for matrix auto-correlation, particle auto-correlation, and matrix–particle cross-correlation. Each basis vector is represented as an image of 500 × 500 = 250,000 pixels. By examining the PC1 basis vector images (leftmost column), a bright circular region is observed at the center of each image. The intensity values at the centers are approximately equal to the volume fractions of the matrix and particles, while the value at the center of the bottom-left image is close to zero. These observations are consistent with the previously discussed characteristics of two-point spatial correlations statistics, indicating that PC1 effectively retains the dominant features of the original data. In contrast, the basis vector images for PC2 and PC3 (middle and right columns) reveal more complex spatial patterns, suggesting that these components capture higher-order structural variations within the microstructure.

**Figure 12:**
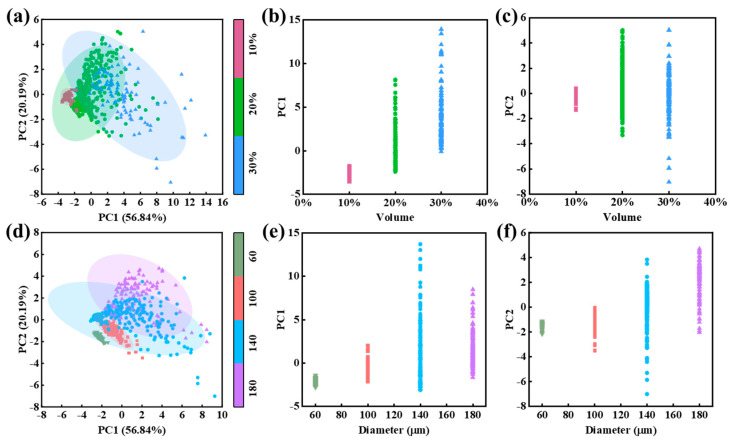
(**a**) Correlation plot of first two PCs (color corresponds to particle volume fractions). (**b**) Correlation plot of particle volume fraction and PC1. (**c**) Correlation plot of particle volume fraction and PC2. (**d**) Correlation plot of first two PCs (color corresponds to particle size). (**e**) Correlation plot of particle size and PC1. (**f**) Correlation plot of particle size and PC2.

PC score maps, obtained by aggregating the first two PC weights of all 600 RVEs, are shown in Figure 12a,d. As observed, samples with different particle volume fractions exhibit significant variation along the PC1 axis, while samples with different particle sizes show greater variation along the PC2 axis. This indicates that PC1 primarily captures structural variations associated with particle volume fraction, whereas PC2 reflects changes related to particle size.

To further explore the intrinsic relationships between the PCs and RVE structural parameters, scatter plots of PC1 and PC2 versus the particle volume fraction and particle size are provided in Figure 12b,c,e,f. A closer examination reveals that, as the particle volume fraction varies, the sample data points show a much clearer separation along the PC1 dimension than along PC2. Conversely, with changes in the particle size, the distribution of data points along PC2 is more distinct than that along PC1. These observations further support the findings in Figure 12a,d.

### 3.3. Machine Learning Methods

#### 3.3.1. Machine Learning Models

Given the nonlinear relationship between the microstructure characteristics and macroscopic properties after dimensionality reduction, this study adopted five different methods for model construction. Through comparative analysis, the optimal method was identified to improve.

(1) XGBoost

**Figure 13:**
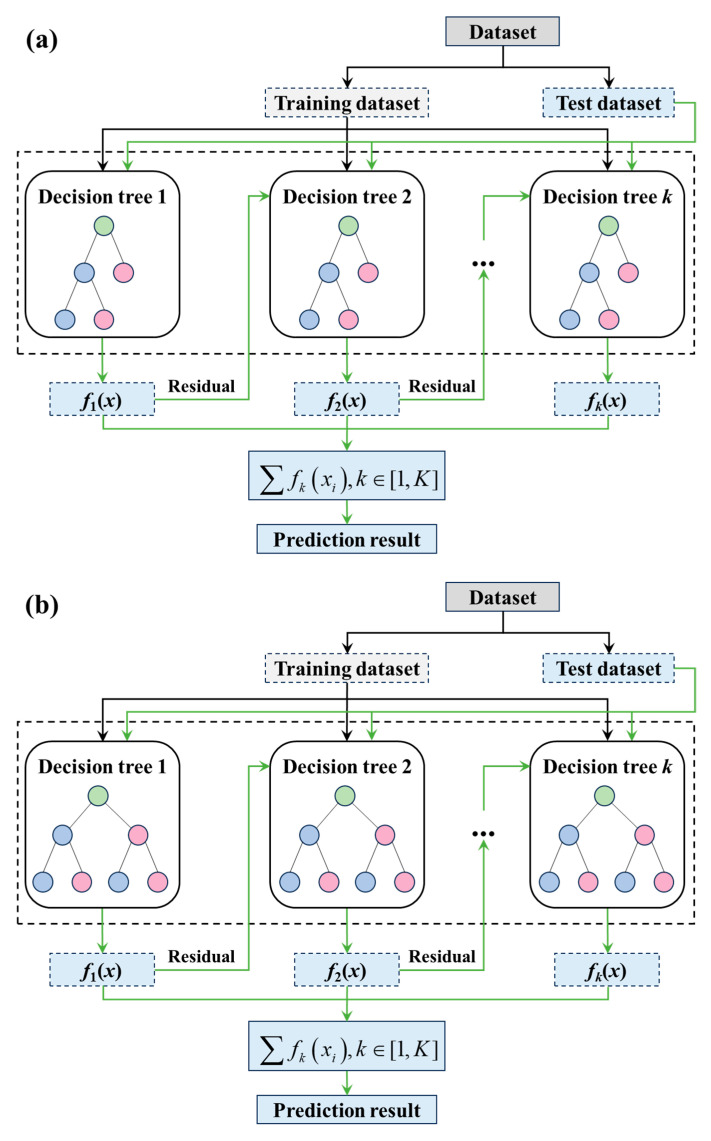
Workflow diagrams of machine learning algorithms. (**a**) XGBoost. (**b**) CatBoost.

XGBoost [64] is based on the gradient boosting decision tree (GBDT) method. It fits the data by iteratively building decision trees, and in each iteration, the prediction is improved by minimizing the residuals. The goal of each new tree is to correct the errors made by the previous tree, thereby progressively enhancing the model’s prediction accuracy. The workflow of the XGBoost algorithm is illustrated in Figure 13a.

(2) CatBoost

Compared with XGBoost, CatBoost [65] is specifically optimized for handling categorical features. Unlike traditional methods that require converting categorical variables into one-hot encodings, CatBoost processes categorical data directly using an ordered boosting technique, which reduces information loss and enhances both the efficiency and accuracy of the model. The workflow of the CatBoost algorithm is illustrated in Figure 13b.

(3) Random forest (RF)

**Figure 14:**
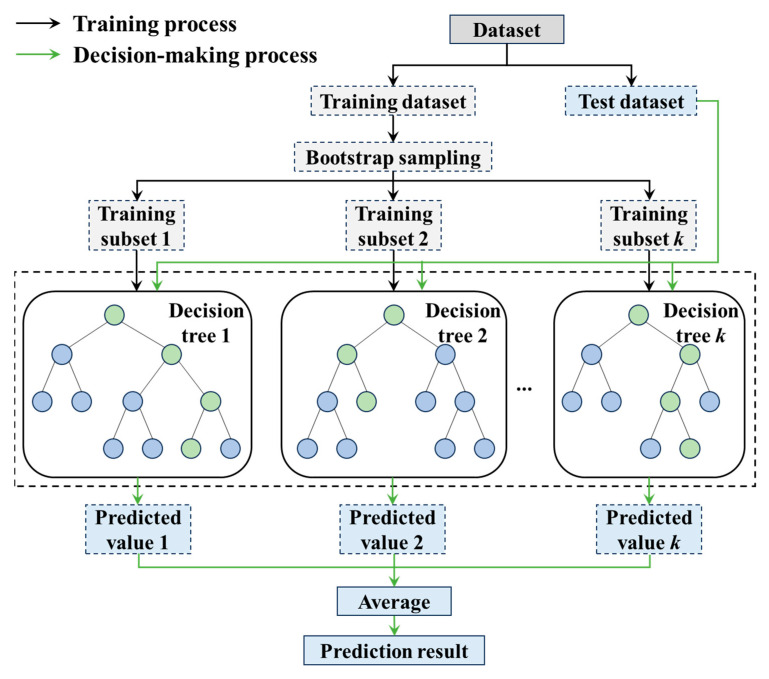
Workflow diagram of RF algorithm.

RF [66] is an ensemble learning method that improves predictive performance and model stability by training multiple independent decision trees and averaging their predictions. During training, each tree is constructed using bootstrap sampling and random feature selection, which increases diversity among the trees and reduces the risk of overfitting. The workflow of the RF algorithm is illustrated in Figure 14.

(4) Support vector regression (SVR)

**Figure 15:**
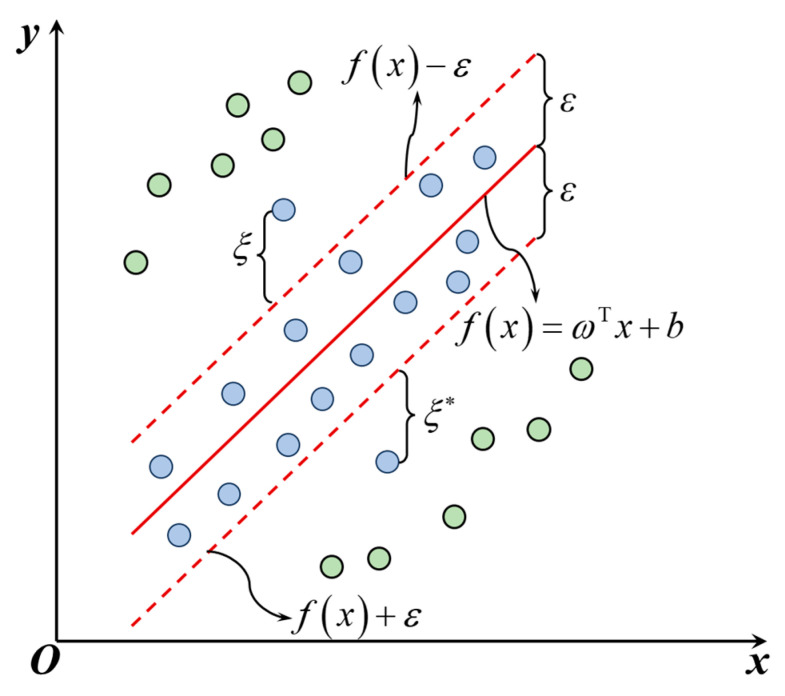
Schematic illustration of SVR principle.

The basic principle of SVR is to employ a kernel function to map nonlinear data from a low-dimensional space to a high-dimensional space, where the data become linearly separable. An optimal hyperplane is then determined to minimize the distance from the farthest sample points to the hyperplane, as illustrated in Figure 15. The SVR problem can be described as follows:

(7) $$f(x)=w^{T}x+b$$

(8) $$\underset{\omega ,b,\xi_{i},{\hat{\xi }}_{i}}{\min}\frac{1}{2}{\left\|\omega \right\|}^{2}+C\sum_{i=1}^{m}\left(\xi_{i}+\xi_{i}^{*}\right)$$

(9) $$\mathrm{Subject}\;\mathrm{to}\;y_{i}-f\left(x_{i}\right)\leq \varepsilon +\xi_{i},\;\xi_{i}\geq 0$$

(10) $$f\left(x_{i}\right)-y_{i}\leq \varepsilon +\xi_{i}^{*},\;\xi_{i}^{*}\geq 0$$

where *w* denotes the weight vector, *b* is the bias term, ξ and $\xi^{*}$ are slack variables, *y_i_* represents the predicted value, *ε* is the insensitive loss parameter, and *C* is the penalty coefficient. The choice of kernel function significantly affects the fitting capability and computational efficiency of the SVR model. In this study, both the polynomial (Poly) kernel and the radial basis function (RBF) kernel are employed.

#### 3.3.2. Hyperparameter Optimization

The performance of machine learning models is highly dependent on the selection of hyperparameters. However, the hyperparameter space is complex, and the search process is extremely time-consuming, requiring substantial computational resources for model training. Moreover, due to the lack of a direct mathematical relationship between hyperparameters and model performance, traditional gradient-based optimization methods are often ineffective.

**Figure 16:**
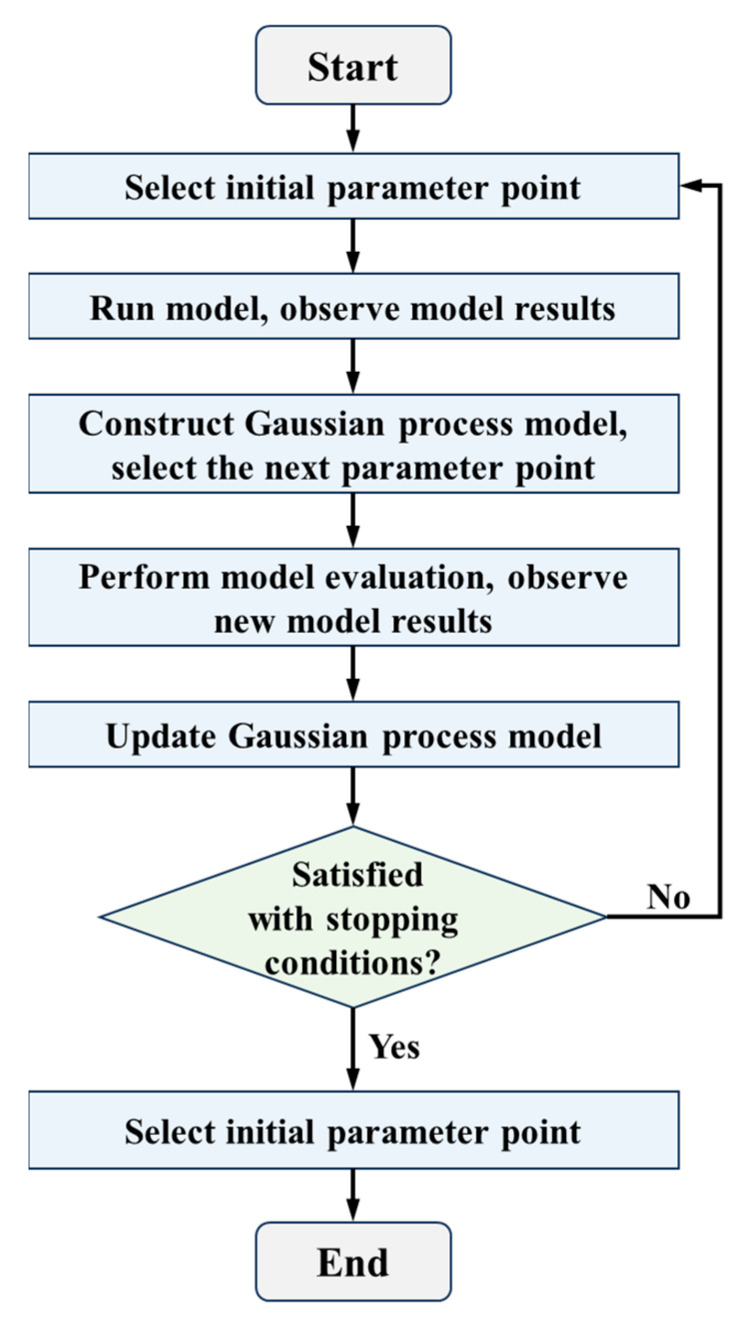
Bayesian optimization flowchart.

To address the aforementioned issues, this study introduces the Bayesian optimization (BO) algorithm [67]. By constructing a surrogate model of the objective function and using a small number of evaluated hyperparameter points for fitting and prediction, BO significantly reduces the number of training iterations. Compared to traditional methods such as random search [68], BO is more efficient in exploring the hyperparameter space and substantially shortens computation time. Furthermore, its inference mechanism, based on observed values, does not require explicit gradient information, making it effective in handling the non-differentiability of the objective function. The core idea of BO is to construct a probabilistic model of the objective function (typically a Gaussian Process model) to infer the shape of the objective function and then select the parameter points most likely to improve the objective function value, thus progressively optimizing the function. The workflow of BO is illustrated in Figure 16.

## 4. Results and Discussion

### 4.1. Extraction and Validation of SP Linkages

The previous sections introduced the methodology for constructing SP linkages. In this section, the effectiveness of the proposed method is validated using the dataset. Specifically, 80% of the dataset is randomly selected as the training set, while the remaining 20% is reserved as the independent test set. To ensure model robustness and generalization, 5-fold cross-validation is conducted within the training set. The cross-validation results are used to optimize the model’s hyperparameters and evaluate predictive performance prior to final testing. The hyperparameter space settings for the machine learning models are summarized in Table 2.

**Figure 17:**
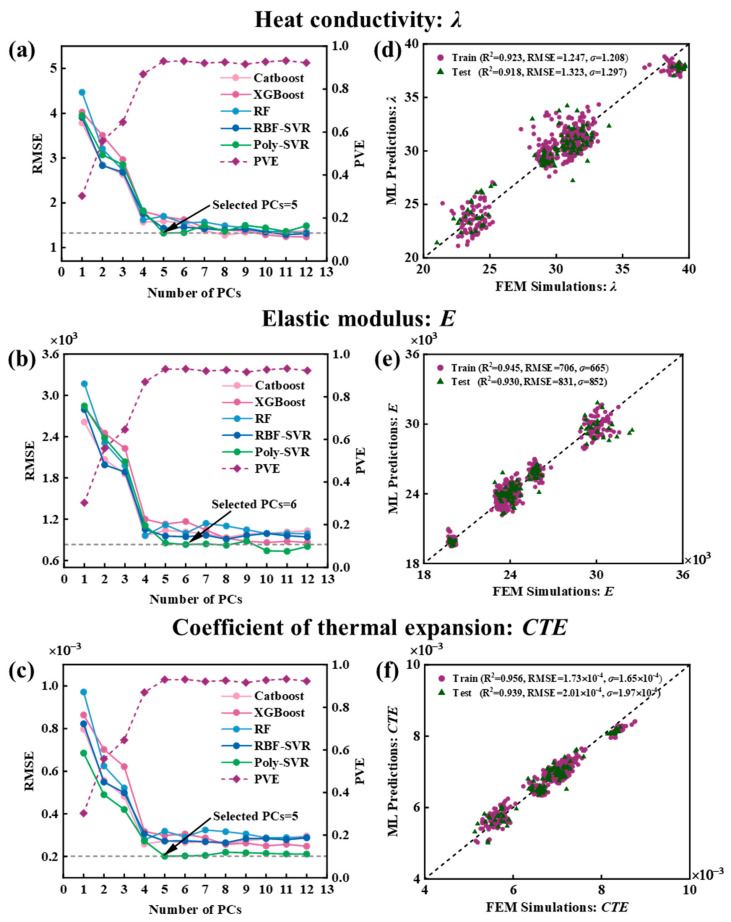
RMSE of different machine learning models predicting (**a**) thermal conductivity, (**b**) elastic modulus, and (**c**) CTE under varying numbers of PCs; comparison between Poly-SVR model predictions and simulation results for (**d**) thermal conductivity, (**e**) elastic modulus, and (**f**) CTE.

Figure 17a–c show the RMSE and PVE of different machine learning models in predicting the thermal conductivity, elastic modulus, and CTE of CPRMMCs under varying numbers of PCs. As shown, when the number of principal components is fewer than five, the RMSE decreases significantly, while the PVE increases sharply. This indicates that a small number of principal components can already capture the dominant features of the data, leading to a substantial improvement in model prediction performance. It also confirms that PCA dimensionality reduction preserves enough useful information to improve the predictive performance of the model. However, when the number of principal components exceeds five, the decrease in the RMSE becomes more gradual, and the growth of PVE plateaus, with only marginal improvements. This suggests that including more features does not significantly enhance the model’s predictive power and may instead introduce redundancy, increasing model complexity and raising the risk of overfitting. Among all evaluated models, the Poly-SVR model exhibits the best predictive performance for thermal conductivity, elastic modulus, and CTE when the number of principal components is 5, 6, and 5, respectively.

To further verify the accuracy of the constructed Poly-SVR model, Figure 17d–f compare the predicted results of the Poly-SVR model with those obtained from finite element simulations for thermal conductivity, elastic modulus, and CTE. The data points are observed to be evenly distributed around the X = Y diagonal, indicating a high level of agreement between the Poly-SVR predictions and FEM results. In addition, the *R*^2^ values for both the training and test sets exceed 0.91, further confirming the accuracy and reliability of the Poly-SVR model in predicting thermodynamic properties.

In the context of material property prediction, the Poly-SVR model demonstrates a significant advantage in computational efficiency while maintaining high predictive accuracy. Traditional finite element methods require approximately one hour to process a single sample, resulting in a total computation time of around 600 h for 600 samples. In contrast, the Poly-SVR model has almost negligible prediction time for each sample after training and can achieve fast and accurate predictions in an extended parameter space. This substantial time-saving advantage highlights the practical value of the Poly-SVR model for material property prediction, particularly in scenarios involving large-scale datasets, where it can greatly enhance computational efficiency.

### 4.2. Discussion

Subsequently, this study systematically analyzes the influence of truncation level and RVE quantity on the predictive performance of the Poly-SVR model. Two sets of comparative conditions are examined: first, the truncation levels of 0, 96, and 146 are tested while keeping the number of RVEs fixed at 600; second, the number of RVEs is reduced from 600 to 300 to further evaluate its effect on model performance.

Table 3 provides a detailed summary of the Poly-SVR model’s prediction results under different truncation levels and RVE quantities. The results show that when the truncation level increases from 0 to 96, the RMSE significantly decreases and the *R*^2^ value increases, indicating improved predictive capability. This suggests that truncation removes part of the redundant information in the two-point statistics, thereby enhancing the distinctiveness of the data features. In contrast, when the truncation level is further increased from 96 to 146, the changes in the RMSE and *R*^2^ become negligible, implying that the central peak of the two-point statistics contains the microstructure information of maximum payload. Furthermore, when the number of RVEs is reduced from 600 to 300, the model shows improved prediction performance for thermal conductivity, negligible change for elastic modulus, and a decline in prediction accuracy for the CTE. This observation further confirms the strong dependency between model effectiveness and dataset size, indicating that under the premise of maintaining sufficient data, an appropriate sample size plays a crucial role in determining the predictive performance of machine learning models. Specifically, the improvement in thermal conductivity prediction with fewer RVEs may be attributed to the model’s ability to focus on more relevant features, reduce overfitting, and avoid noise or less informative data points.

This study proposes an MI-based method to explore the SP linkages of CPRMMCs and successfully carries out mapping between the microstructure and thermodynamic properties using efficient machine learning methods. Compared with traditional experimental and numerical simulation approaches, this method offers significant advantages in both data processing and model construction. The data-driven methodology is capable of capturing the complex nonlinear relationships between material microstructures and their properties while enabling high-efficiency performance prediction. This approach not only saves substantial time and computational costs but also avoids the complexity and uncertainty often encountered in conventional methods. By combining two-point spatial correlation-based graph Fourier transform with PCA, this study effectively extracts representative microstructural features from a high-dimensional feature space. This approach simplifies data processing while retaining the key information necessary for the accurate prediction of material properties. One of its major advantages lies in reducing the risk of model overfitting caused by excessive features while also improving the model’s generalization ability, which refers to its ability to maintain strong predictive performance when applied to new, unseen datasets.

The current approach, however, relies on traditional machine learning methods to analyze numerical simulation data for six parameter combinations. Future research could explore more advanced techniques [30], such as convolutional neural networks, graph neural networks, and symbolic regression, and extend the analysis to simulations or experiments involving a broader range of parameter combinations, including factors such as particle shapes, orientations, spatial distributions, and additional processing parameters. Additionally, future studies could incorporate supplementary data sources, such as electron microscopy [69] and X-ray tomography, to further enhance prediction accuracy and reliability.

## 5. Conclusions

This study proposes an efficient and accurate approach within the MI framework for establishing structure–property linkages between the microstructure and thermodynamic properties of CPRMMCs. It integrates the FEM, graph Fourier transform, PCA, and machine learning methods. By combining graph Fourier transform based on two-point spatial correlations with PCA, we successfully reduced the high-dimensional microstructure data (750,000 dimensions) to just five to six key PCs. This dimensionality reduction preserved the core features of the microstructure, such as particle volume fraction, particle size, and spatial distribution. Using these PCs, precise mapping between the microstructure and thermodynamic properties was carried out with a Bayesian-optimized Poly-SVR model. The results show that this model exhibits excellent prediction accuracy, achieving efficient predictions with only a few microstructure features, and all *R*^2^ values exceed 0.91. This confirms its effectiveness and reliability in predicting material properties. Future research will focus on integrating multi-source data to provide more accurate support for materials design and optimization.

## Figures and Tables

**Table 1:** Material properties of UO_2_, Zr, and interface [53,54].

Material	Density (kg/m^3^)	Thermal Conductivity W/(m·K)	Coefficient of Thermal Expansion (°C^−1^)	Elastic Modulus (MPa)	Poisson’s Ratio
UO_2_	10,600	2.3026	1.54 × 10^−5^	168,137	0.316
Zr	6550	47.4328	8.95 × 10^−3^	15,150	0.34
Interface	9790	11.3289	1.80 × 10^−3^	137,540	0.3208

**Table 2:** Hyperparameter settings for machine learning models.

Machine Learning Model	Hyperparameter Settings
Poly-SVR	*C* ∈ [0.1, 100], epsilon ∈ [0.01, 0.5], degree ∈ [2, 5], coef0 ∈ [0, 1]
RBF-SVR	*C* ∈ [0.1, 100], epsilon ∈ [0.01, 0.5], gamma ∈ [0.01, 0.1, 1]
RF	n_estimators ∈ [10, 100], max_depth ∈ [3, 10], min_samples_split ∈ [2, 20], min_samples_leaf ∈ [1, 20]
XGBoost	n_estimators ∈ [10, 100], max_depth ∈ [3, 10], learning_rate ∈ [0.01, 0.5], subsample ∈ [0.6, 1], colsample_bytree ∈ [1, 20]
CatBoost	Iterations ∈ [10, 100], depth ∈ [3, 10], learning_rate ∈ [0.01, 0.5], l2_leaf_reg ∈ [1, 10], subsample ∈ [0.6, 1], colsample_bylevel ∈ [0.6, 1]

**Table 3:** Prediction results of Poly-SVR model with different truncation levels and RVE quantities.

Property	Truncation	Data Volume	No. RVEs	Training RMSE	Test RMSE	Training *R*^2^	Test *R*^2^
Thermal conductivity	0	1,436,592	600	1.292	1.681	0.918	0.867
	96	750,000	600	1.247	1.323	0.923	0.918
	96	750,000	300	1.182	1.227	0.930	0.935
	146	480,000	600	1.231	1.379	0.925	0.911
Elastic modulus	0	1,436,592	600	701.795	1156.328	0.946	0.865
	96	750,000	600	706.179	830.8523	0.945	0.930
	96	750,000	300	696.629	810.255	0.948	0.929
	146	480,000	600	731.614	892.195	0.941	0.920
Coefficient of thermal expansion	0	1,436,592	600	0.000229	0.000327	0.951	0.897
	96	750,000	600	0.000216	0.000252	0.956	0.939
	96	750,000	300	0.000291	0.000326	0.918	0.905
	146	480,000	600	0.000228	0.000251	0.951	0.939

## Data Availability

The original contributions presented in this study are included in the article. Further inquiries can be directed to the corresponding author.
